# HOX Gene Aberrant Expression in Skin Melanoma: A Review

**DOI:** 10.1155/2012/707260

**Published:** 2012-10-02

**Authors:** Gérald E. Piérard, Claudine Piérard-Franchimont

**Affiliations:** Department of Dermatopathology, Liège University Hospital, 4000 Liège, Belgium

## Abstract

The homeobox family and its subset of HOX gene products represent a family of transcription factors directing DNA-protein and protein-protein interactions. In the embryo, they are central regulators in cell differentiation during morphogenesis. A series of genes of the four HOX gene clusters A, B, C, and D were reported to show aberrant expressions in oncogenesis, particularly in cutaneous malignant melanoma (CMM). They are involved in cell proliferation and progression in the CMM metastatic path. We present relevant peer-reviewed literature findings about the aberrant expression of HOX genes in CMM. The number of CMM cell nuclei exhibiting aberrant HOX protein expression appears correlated with tumour progression.

## 1. Introduction

Primary cutaneous malignant melanoma (CMM) is an increasingly frequent malignancy in the White population of western countries [1]. The neoplasm arises from melanocytes in a complex process. In each CMM, the tumour size is closely associated with cell proliferation including the size of the growth fraction [2, 3]. In recent years, some progress has been reached in the understanding of primary genetic alterations involved in the CMM initiation [4–6]. The following neoplastic progression is accompanied or supported by other additional genetic mutations. Many oncogenes, tumour suppressor genes, and altered signalling pathways have been identified [7–11]. Thus, following the initial malignant thigmotropism step [12], a variety of subsequent mutations occur promoting the CMM invasiveness [7]. Some of these changes are probably involved in the rise of potential for metastasis. The malignant cells prone to metastasize but still in contact with the primary tumour are currently not identifiable under the microscope.

 Although some specific CMM subtypes are clinically and histopathologically distinct, these features do not commonly exhibit independent prognostic value. Despite a wealth of information, the prognostic indicators for the progression of primary CMM currently remain the tumour depth and proliferation, as well as the presence or absence of ulceration and micrometastases [13]. Several key molecular pathways are involved in the CMM initiation and progression. The diversity of CMM presentations allows to establish a classification providing insight into CMM epidemiological data and their molecular counterparts [14, 15]. In addition, the translation of CMM molecular biology to relevant clinical correlates and novel therapies has made significant progress over the past few years [6, 9, 10].

 Gene expression profiling of human cancers, in particular CMM, allows for a unique insight into the genes intimately involved in the neoplastic process. Clinical and genomic evidence suggests that the metastatic potential of a primary CMM may be dictated by prometastatic events that have additional oncogenic capability [16]. The homeobox gene family and its subset represented by HOX genes, and their related proteins represent, a cluster of molecules that are likely involved in the potential for metastasis expressed by some cancer cells including CMM cells [17]. The homeodomain is a four-*α*-helix helix-loop-helix DNA binding motif, and its flanking sequences provide either activating or repressing functions for target gene transcription [18, 19]. These homeogenes are organized in four multigene clusters named A, B, C, and D mapped on chromosomes 2, 7, 12, and 17 [20]. Each cluster is composed of 9 to 11 HOX genes. Any HOX gene is part of a family of DNA sequences involved in a series of transcription factors affecting tissue development and cell differentiation. HOX proteins are present in nuclei and they direct DNA-protein and protein-protein interactions. 

 The most common mutation in human CMM BRAF (V600E) activates the serine/threonine kinase BRAF and causes excessive activity in the mitogen-activated protein kinase (MAPK) pathway [6]. BRAF (V600E) mutations are also present in benign melanocytic naevi, highlighting the importance of additional genetic alterations in the genesis of malignancies [21]. Such changes include recurrent copy number variations that result in the amplification of oncogenes. For certain amplifications, the large number of genes in the interval has precluded an understanding of the cooperating oncogenic events.

 The present coverage of peer-reviewed articles revisits the salient aspects of aberrant expression of HOX genes in human CMM.

## 2. HOX Landscape in CMM

HOX genes were originally discovered in *Drosophila melanogaster*. They appear highly conserved during evolution from simple organisms to vertebrates including humans [18, 19]. In humans, HOX proteins are crucial for skin morphogenesis, involved in the control of axial patterning, and responsible for hereditary malformations and some cancers including CMM. During ontogenetic development HOX gene expression appear-tumour-stage related and tissue or region specific. Their relative position on chromosomes is connected to their expression domains along the anterior-posterior axis of the central nervous system. In addition, HOX genes coding for transcription factors is involved in the process of neoplastic progression including CMM [18–22]. Indeed, aberrant HOX expression is typically associated with oncogenesis. They vary according to the histopathologic type and progression stage of the neoplasm including metastasis. The location of the primary CMM on the body is unrelated to the aberrant HOX expression.

### 2.1. HOX-A Locus

HOX-A1 is an oncogene playing a pivotal role in human cancers by stimulating cell proliferation, anchorage-independent cell growth, and loss of contact inhibition [23]. In addition, HOX-A1 and -A2 expression was higher in CMM with distant metastases compared to CMM without metastases [16, 22]. Of note, HOX-A5 downregulates angiogenesis by mitigating the activity of proangiogenic genes and upregulating the expression of antiangiogenic genes [24]. HOX-A9 was shown to be epigenetically silenced in CMM [25]. The expression of HOX-A11 and -A13 was reported to be higher in CMM than in melanocytic naevi [22]. HOX-A11 expression was further more altered in metastatic CMM than in the pT1-T3 nonmetastatic tumours. 

### 2.2. HOX-B Locus

HOX-B9 aberrant expression was reported to be higher in CMM than in melanocytic naevi [22]. HOX-B2 expression was higher in metastatic CMM than in pT1-T3 nonmetastatic lesions. HOX-B13 appeared overexpressed in CMM with distant metastases than in CMM without metastases [26]. HOX-B7 was reported to be overexpressed in proliferative CMM cells [27, 28]. The bone morphogenetic protein (BMP)-4 is overexpressed in CMM cells leading to the induction of basic fibroblast growth factor (bFGF) mediating CMM cell migration. This biological pathway starts with the reduced expression of the micro-RNA miR-196 in CMM cells compared to nonneoplastic melanocytes. It is particularly activated by increasing HOX-B7 transcription factor levels [27, 28]. An inverse relationship was established in the patterns of expression of HOX-B7 and the promyelocytic leukemia zinc finger (PLZF) protein, which acts as a suppressor. Hence, HOX-B7 and PLZF are functionally independent and their coupled deregulation may account for most if the alterations found in CMM [29].

### 2.3. HOX-C Locus

The HOX-C locus plays a role in the CMM metastatic phase. There is an aberrant expression of genes located near the HOX-C locus corresponding to HOX-C10, C11, and C13. It results in alterations in integrins, ICAM-1, N-Ras, IL-1A, IL-6, and TNF-*α* [30]. HOX-C4 was apparently overexpressed in CMM with distant metastases compared to CMM without metastases [22]. HOX-C13 aberrant expression was higher in metastatic CMM compared to pT1-T3 nonmetastatic tumours [22, 31]. MicroRNA (miRNA)-196a appeared to negatively regulate the expression of the transcription factor HOX-C8 [32, 33].

### 2.4. HOX-D Locus

HOX-D3 is involved in regulating cell-cell interactions in CMM, as well as the motile and invasive behaviours of CMM cells [34]. By contrast, nonneoplastic melanocytes do not express HOX-D3. HOX-D12 and -D13 were reported to be expressed in CMM at a higher level than in melanocytic naevi [22, 33]. This finding is in line with a study on 79 tumour tissue types in which significant differences were reported between specific normal tissues and the corresponding neoplasms exhibiting an increase in HOX-D13 expression [35].

## 3. Discussion

Compared to primary CMM, their metastases commonly express higher levels of genes including MAGE, GPR 19, BCL2A1, MMP14, SOX5, BVB1, and RGS20 [32]. The same is true for the presently gathered series of HOX gene disturbances. The transition from non-metastatic to metastatic expression of HOX activation levels occurs as the CMM increases in thickness. The transition in gene expression appears to occur at different thicknesses for different CMM genes. This critical transition timing for the emergence of the metastatic phenotype is a key moment in the evolution of CMM [36]. Multiple genes are involved in the progression or suppression of the metastatic CMM phenotype. Tumour oncogenes include SPP-1, MITF, CITED-1, GDF-15, c-Met, and HOX loci [37]. Suppressor genes include PITX-1, CST-6, PDGFRL, DSC-3, POU2F3, CLCA2, and ST7L. Silencing oncogenes and tumour suppressor genes is possible [25].

 HOX genes are transcriptional regulators, which modulate embryonic morphogenesis and pathological tissue remodelling in adults via regulation of genes associated with cell-cell or cell extracellular matrix (ECM) interactions [24]. Members of the HOX family of homeodomain-containing transcription factors are deregulated in CMM [38]. HOX proteins are transactivating factors regulation gene expression. The homeodomain is capable to bind to specific DNA sequences, including promoters of other HOX genes, and to enhance or inhibit transcription. Alteration of such a complex network controlling cell proliferation contributes to the multistep process underlying the onset of neoplasia. Indeed, alterations of specific groups of HOX genes are associated with neoplastic transformation in different tissues including the breast, kidney, lymphoid organ, colon, lung, thyroid, bladder, prostate, and skin [17]. Accordingly, identifying the HOX expression levels is expected to have a diagnostic value and to establish the prognosis of the neoplasm under consideration. Different specific HOX gene aberrant expressions have been reported in CMM. It has been suggested that HOX genes contribute to CMM transformation and progression via the basic fibroblast growth factor (bFGF), which promotes an autocrine loop in CMM and is one of the major angiogenic factors [27]. Initiation of CMM metastasis is a key event in the neoplastic progression. The molecular mechanisms and the possible genetic dysfunction underlying this process remain obscure, but the intervention of HOX genes is likely.

## 4. Conclusion

The gene expression profiling of primary, nonmetastatic and metastatic CMM has resulted in the identification of several genes that are centrally involved in the progression and metastatic potential of CMM [7]. Among these mechanisms, homeoproteins represent transactivating factors issued from HOX genes that regulate a series of gene expression [18, 19]. Some alterations in specific HOX genes are activated during various malignant transformations. 

The regulatory function of HOX genes is possibly mediated by crucial growth factor genes. After the identification of the genes encoding several adhesion molecules, such as cytotactin [39], N-CAM [40, 41], and L-CAM [42], as target genes for homeoproteins [43], it was shown that bFGF was the first growth factor involved in the complex network underlying HOX gene effects on cell proliferation and differentiation [44]. The autocrine bFGF production by CMM cells sustains tumoural growth as an early event in CMM progression. Other autocrine loops are likely involved in the same process and implicate diverse cytokines, growth factors, and receptors [27]. HOX activation possibly boosts the rate of proliferation of CMM or represents a critical step in the progression and metastatic processes. 

The intervention of HOX gene dysfunction in the metastatic CMM switch is supported by the absence of such altered function in melanocytic naevi and primary non-metastatic CMM. The search for new molecular markers that allow to select the tumour cell clones with respect to their aggressiveness and their ability to metastasize is certainly one of the most important future goals in CMM biomedical research. In such a context, the HOX genes represent ideal therapeutic targets. Several molecules, such as antisense oligonucleotide (ASO) and interfering RNA (iRNA), were shown to be possibly capable to interact with crucial genes involved in the altered proliferation of the neoplastic cells [34, 45].
